# A Conservative Amino Acid Mutation in the Master Regulator FleQ Renders *Pseudomonas aeruginosa* Aflagellate

**DOI:** 10.1371/journal.pone.0097439

**Published:** 2014-05-14

**Authors:** Ruchi Jain, Barbara I. Kazmierczak

**Affiliations:** 1 Department of Internal Medicine (Infectious Diseases), Yale University School of Medicine, New Haven, Connecticut, United States of America; 2 Department of Microbial Pathogenesis, Yale University School of Medicine, New Haven, Connecticut, United States of America; The Scripps Research Institute and Sorrento Therapeutics, Inc., United States of America

## Abstract

Flagellar-based motility plays a critical role in *Pseudomonas aeruginosa* pathogenesis, influencing both the establishment of bacterial infection and the host's response to the pathogen. Nonetheless, aflagellate clinical strains are often isolated from acutely and chronically infected patients and include the virulent laboratory strain PA103. We determined that PA103's aflagellate phenotype is the result of a single amino acid change (G240V) in the master flagellar regulator, FleQ. This mutation, which lies just outside the Walker B box of FleQ, abrogates the ability of FleQ to positively regulate flagellar gene expression. Reversal of this seemingly conservative amino acid substitution is sufficient to restore swimming motility to PA103, despite the presence of mutations in other flagellar genes of PA103. We also investigated the consequences of restoring flagellar assembly on PA103 virulence. Although a negative correlation between flagellar assembly and Type 3 secretion system (T3SS) expression has been reported previously, we did not observe downregulation of T3SS expression or function in Fla+ PA103. Restoration of flagellar assembly did, however, amplify IL-1 signals measured during murine pulmonary infection and was associated with increased bacterial clearance. These experiments suggest that loss of flagellar motility may primarily benefit PA103 by attenuating pathogen recognition and clearance during acute infection.

## Introduction

*Pseudomonas aeruginosa* is a Gram-negative human pathogen that causes acute and chronic infections in individuals with compromised local barrier defenses (e.g. burns, corneal ulcers, Cystic Fibrosis (CF)), or systemic immunocompromise [1]. Bacterial motility is crucial for *P. aeruginosa* attachment to and colonization of environmental reservoirs, as well as of host tissues during infection. *P. aeruginosa* exhibits three distinct types of motility, which are powered by flagella (swimming) [2], Type IV pili (twitching) [3] or both motility organelles (swarming) [4]. Flagella and pili also contribute to the formation and maturation of biofilms, which are relatively resistant to clearance by antibiotics and phagocytic cells *in vitro* and *in vivo* [5], [6].

The innate immune system recognizes bacterial flagellin via both extra- and intracellular pathogen recognition receptors, leading to the activation of host defense mechanisms that favor bacterial clearance [7]–[10]. Thus it is not surprising that many bacteria, including *P. aeruginosa*, downregulate flagellar expression in the host environment [11], [12]. Downregulation of flagellar gene expression results from neutrophil elastase-mediated degradation of the hook-basal-body FlgE protein and subsequent intracellular accumulation of the anti-sigma factor FlgM [13].

Flagellar gene expression in *P. aeruginosa* is under the control of a master transcriptional regulator, FleQ [14]. FleQ is an enhancer binding protein (EBP) that belongs to the NtrC family of bacterial transcriptional factors. FleQ contains an N-terminal FleQ domain, a central AAA+/ATPase σ^54^-interaction domain and a C-terminal helix-turn-helix DNA-binding domain [15]. FleQ activates transcription of genes involved in flagellar export (*flhA*, *fliLMNOPQ*) and localization (*flhF*, *fleN*), as well as structural components of the flagellar basal body (*fliEFG*) and the FleSR two-component system in a σ^54^-dependent manner [16]. FleQ also binds the *pel* and *psl* operon promoters, where it activates or represses the expression of these exopolysaccharide biosynthetic operon in the presence or absence of cyclic-di-GMP, respectively, in σ^54^-independent manner [17]. Thus FleQ activity is implicated in the control of loci important for both motile and sessile phenotypes.

FleQ exhibits ATPase activity [18], which is thought to provide energy for EBP remodeling of the σ^54^-RNA polymerase closed complex and subsequent loading of DNA template into the active site of the RNA polymerase [15]. Recent work shows that cyclic-di-GMP inhibits FleQ ATPase activity, most likely by competing with ATP for binding to the Walker A box [18]. Inhibition is enhanced when FleQ is in complex with another flagellar regulator, FleN [18], and this likely accounts for earlier observations that FleN functions as a FleQ anti-activator *in vivo* [19], [20].

Most environmental and clinical isolates exhibit flagellar motility, but inactivating mutations in flagellar genes, among them FleQ, are observed in sequenced isolates from chronically infected CF patients [21]–[23]. Non-flagellated strains isolated from acutely infected non-CF patients are also described and include PA103, a well-studied serogroup O11 strain initially isolated from a human pneumonia patient [24]. Loss of virulence factor expression by clinical isolates is often due to mutations in master regulators. This was described for Type 3 secretion system (T3SS)-negative *P. aeruginosa* strains isolated from CF patient airways [23]. Over-expression of the T3SS transcriptional activator, ExsA, was able to restore a functional T3SS to these isolates, demonstrating that the remainder of this complex protein secretion system was intact [25]. We tested whether an analogous loss of transcriptional activator function might account for the aflagellate phenotype of PA103.

## Results

### PA103 expresses a mutant FleQ protein that does not support flagellar assembly

**Figure 1:**
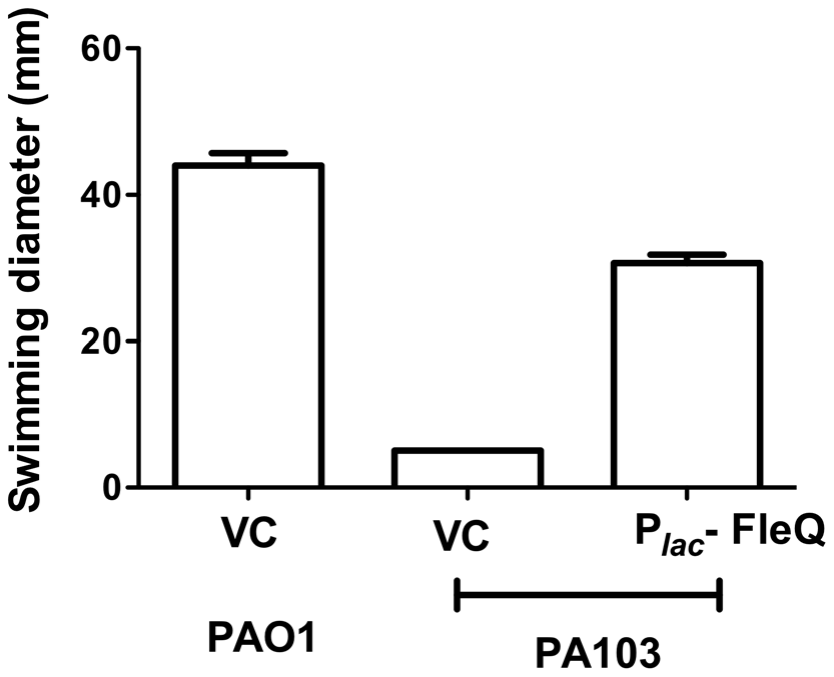
Expression of FleQ_PAO1_ is sufficient for PA103 to swim. Swimming zone diameters were measured 16% LB agar plates. Bars represent mean ± S.D. of three replicates. VC: vector control.

PA103, a well-studied *P. aeruginosa* strain originally isolated from a patient with acute pneumonia, does not express flagella [26], but the basis for its aflagellate phenotype is not known. We hypothesized that PA103 lacked FleQ expression or function, and tested this by amplifying and cloning the *fleQ* gene from the flagellated reference strain PAO1 and expressing it in PA103. PA103 bacteria expressing FleQ_PAO1_ swam, while PA103 bacteria transformed with empty vector (VC) did not (Figure 1). This suggested that the flagellar motility genes controlled by FleQ were present and encoded functional proteins in PA103.

**Figure 2:**
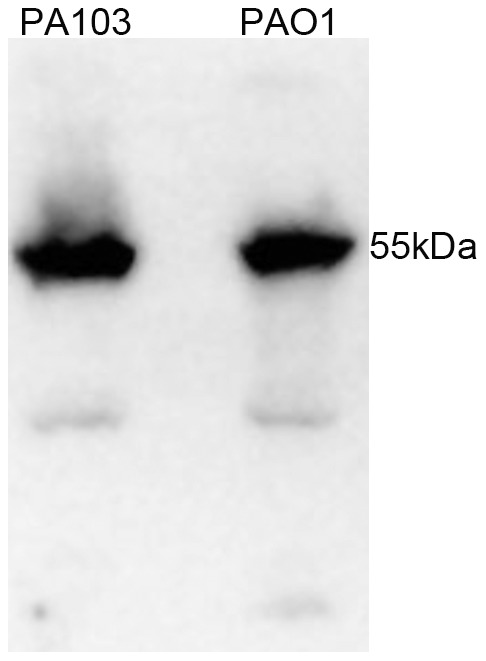
FleQ is expressed in both PAO1 and PA103. Whole cell lysates of PAO1 and PA103 strains (normalized to total protein) were separated by SDS-PAGE and immunoblotted with anti-FleQ antiserum.

Since PA103 swimming motility could be rescued by expression of FleQ_PAO1_, we tested whether PA103 was defective in expression of endogenous FleQ. *fleQ* mRNA levels were assayed in exponential phase PA103 and PAO1 by quantitative RT-PCR. The *fleQ* gene was expressed by both strains, although *fleQ* mRNA levels were 6±2-fold higher in PAO1 as compared to PA103. FleQ protein was detected at similar steady state levels in whole cell lysates prepared from exponential phase PA103 and PAO1 by Western blotting with anti-FleQ antiserum (Figure 2).

We sequenced the *fleQ* gene from PAO1 and PA103 and identified only one missense mutation in *fleQ*_PA103_ (G718T) leading to an amino acid substitution of Gly240Val, in addition to multiple silent mutations (T194G, A215G, T407C, T642C, T662C, T809C, C833A, T1010C, A1046G, C1379T, C1400T, A1415G and T1448C). Glycine_240_ lies just before the putative Mg^++^ binding sequence (hhhhDE) of the Walker B motif of FleQ (Figure S1A). We aligned FleQ sequences from different *Pseudomonas* spp. (Figure S1B) as well as homologs from other flagellated bacteria (Figure S1C), and found that Glycine_240_ was conserved in all sequences except that of FleQ_PA103_.

To complete our analysis of the flagellar operons in PA103, we sequenced a PA103 genomic DNA library (2×250 bp paired-end) on a MiSeq, generating 696,082 raw reads (348,041,000 bp). De novo assembly with Velvet generated 536 contigs (n_50_ = 214,361 bp; max 595,194 bp) with median 18-fold coverage. Flagellar genes were contained within two contigs of length 179,321 bp (*flgB-fliJ*) and 40,082 bp (*fliK*-*motD*), and followed the same gene organization as PAO1, with the exception of the region between *flgL* and *fliC*. This region contains nine open reading frames predicted to encode glycosylation genes (Table 1); glycosylation islands of variable size appear in this position in many *P. aeruginosa* strains including the commonly studied reference strain PAK [27], which is included in this analysis (Genbank ASWU01000018). We again observed the FleQ(G240V) mutation, as well as missense mutations in other flagellar genes relative to PAO1. These are tabulated in Table 1, and clearly did not prevent flagellar assembly or function. We note that PA103 encodes an a-type flagellin, while PAO1 encodes a b-type flagellin [28]. This difference accounts for the low % identity between the *fliC* and *fliD* genes of PA103 and PAO1.

**Table 1:** Details of flagellar genes in PA103.

Gene name	PA locus ID (PAK locus ID*)	% identity, vis a vis PAO1 (PAK*)	Protein function
*flgB*	PA1077 (PAK_04305)	99% (99%)	flagellar basal-body rod protein
*flgC*	PA1078 (PAK_04304)	100% (100%)	flagellar basal-body rod protein
*flgD*	PA1079 (PAK_04303)	100% (100%)	flagellar basal-body rod modification protein
*flgE*	PA1080 (PAK_04302)	98% (98%)	flagellar hook protein
*flgF*	PA1081 (PAK_04301)	100% (100%)	flagellar basal-body rod protein
*flgG*	PA1082 (PAK_04300)	100% (100%)	flagellar basal-body rod protein
*flgH*	PA1083 (PAK_04299)	99% (99%)	flagellar L-ring protein
*flgI*	PA1084 (PAK_04298)	99% (99%)	flagellar P-ring protein
*flgJ*	PA1085 (PAK_04297)	99% (99%)	Peptidoglycan hydrolase FlgJ
*flgK*	PA1086 (PAK_04296)	88% (99%)	flagellar hook-associated protein FlgK
*flgL*	PA1087 (PAK_04295)	68% (100%)	flagellar hook-associated protein 3
*orfA*	(PAK_04294)	(100%)	Aminotransferase
*orfB*	(PAK_04293)	(100%)	Acyl carrier protein
*orfC*	(PAK_04292)	(99%)	3-oxoacyl-ACP
*orfD*	(PAK_04291)	(86%)	3-oxoacyl-ACP reductase
*orfE*	(PAK_04290)	(99%)	Transferase
*orfF*	(PAK_04289)	(99%)	2Fe-2S ferredoxin
*orfG*	(PAK_04288)	(98%)	Acetyltransferase
*orfH*	(PAK_04287)	(78%)	Methyltransferase
*orfI*	(PAK_04281)	(98%)	O-antigen biosynthesis protein
*fliC*	PA1092 (PAK_04280)	77% (94%)	A-type flagellin
*flaG*	PA1093 (PAK_04279)	41% (100%)	
*fliD*	PA1094 (PAK_04278)	43% (99%)	Flagellar cap protein
*fliS*	(PAK_04277)	(100%)	A-type flagellar protein FliS
*fliS*	PA1095 (PAK_04276)	66% (100%)	Flagellar protein FliS
*fliT*	PA1096 (PAK_04275)	47% (100%)	
*fleQ*	PA1097 (PAK_04274)	99% (99%)	Transcriptional regulator
*fleS*	PA1098 (PAK_04273)	100% (99%)	Two component sensor
*fleR*	PA1099 (PAK_04272)	100% (99%)	Two component response regulator
*fliE*	PA1100 (PAK_04271)	100% (98%)	Hook basal body complex protein
*fliF*	PA1101 (PAK_04270)	100% (99%)	M-ring outer membrane protein precursor
*fliG*	PA1102 (PAK_04269)	100% (100%)	Motor switch protein
*fliH*	PA1103 (PAK_04268)	99% (99%)	Probable assembly protein
*fliI*	PA1104 (PAK_04267)	100% (100%)	flagellum-specific ATP synthase
*fliJ*	PA1105 (PAK_04266)	99% (99%)	flagellar protein
*fliK*	PA1441 (PAK_03919)	97% (97%)	putative flagellar hook-length control protein
*fliL*	PA1442 (PAK_03918)	100% (100%)	Flagella assembly
*fliM*	PA1443 (PAK_03917)	100% (100%)	flagellar motor switch protein
*fliN*	PA1444 (PAK_03916)	100% (99%)	flagellar motor switch protein
*fliO*	PA1445 (PAK_03915)	99% (99%)	flagellar protein
*fliP*	PA1446 (PAK_03914)	100% (100%)	flagellar biosynthetic protein
*fliQ*	PA1447 (PAK_03913)	99% (99%)	flagellar biosynthetic protein
*fliR*	PA1448 (PAK_03912)	99% (99%)	flagellar biosynthetic protein
*flhB*	PA1449 (PAK_03911)	100% (100%)	flagellar biosynthetic protein
*PA1450*	PA1450 (PAK_03910)	99% (99%)	Hypothetical protein
*PA1451*	PA1451 (PAK_03909)	99% (99%)	Hypothetical protein
*flhA*	PA1452 (PAK_03908)	99% (99%)	flagellar biosynthesis protein
*flhF*	PA1453 (PAK_03907)	98% (99%)	flagellar biosynthesis protein
*fleN*	PA1454 (PAK_03906)	100% (100%)	flagellar synthesis regulator
*fliA*	PA1455 (PAK_03905)	100% (100%)	Sigma factor
*cheY*	PA1456 (PAK_03904)	100% (100%)	two-component response regulator
*cheZ*	PA1457 (PAK_03903)	100% (100%)	Chemotaxis protein
*PA1458*	PA1458 (PAK_03902)	99% (99%)	Probable two component sensor (CheA)
*PA1459*	PA1459 (PAK_03901)	99% (99%)	Chemotaxis-specific methyltransferase (CheB)
*motC*	PA1460 (PAK_03900)	100% (99%)	Motor protein
*motD*	PA1461 (PAK_03899)	99% (99%)	Motor protein

*PAK sequences from Genbank accession ASWU01000018.

### Mutation of Valine240 to Glycine in FleQ_PA103_ is sufficient to restore flagellar expression and swimming to PA103

**Figure 3:**
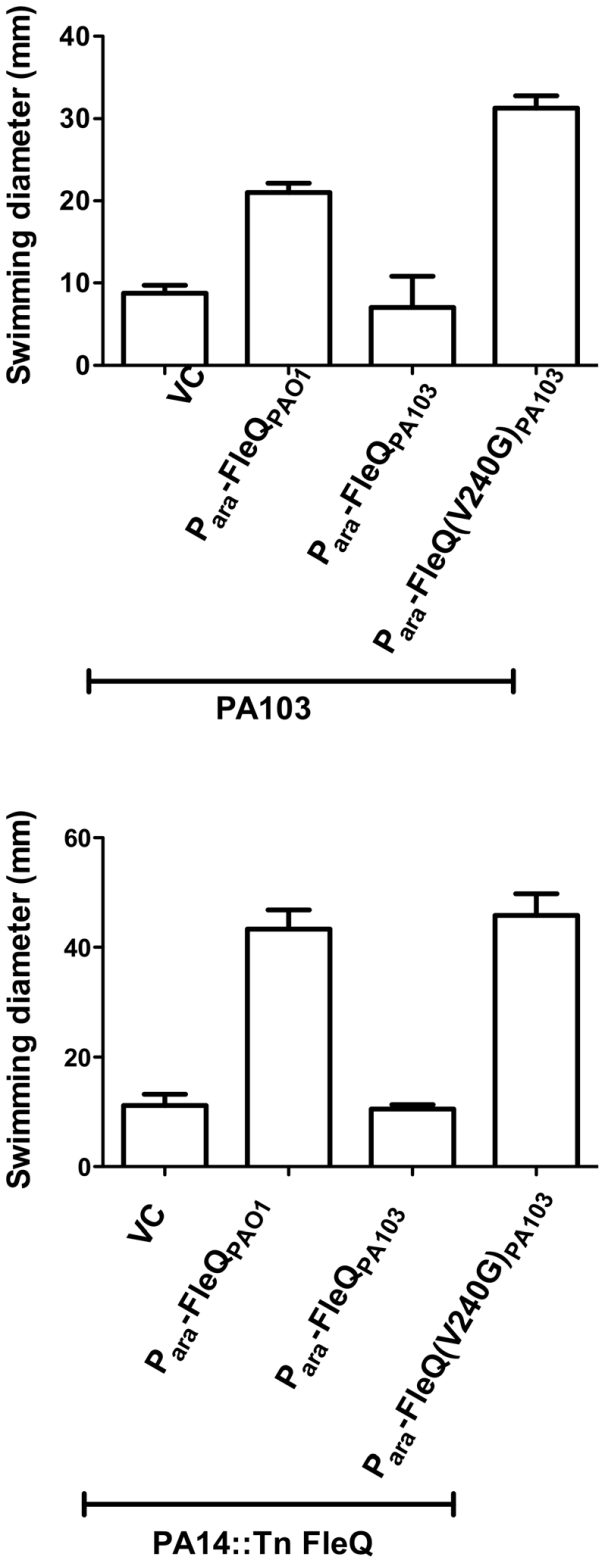
Reversion of the Valine240 mutation to Glycine in FleQ_PA103_ restores FleQ function. (A) Swimming zones were measured for PA103 carrying pMQ72 (VC), or plasmids encoding FleQ_PAO1_, FleQ_PA103_, or the “corrected” allele FleQ(V240G)_PA103_ 16 hr after inoculating 0.3% LB agar plates containing gentamicin and 0.2% arabinose. All genes are expressed from an arabinose inducible promoter (P*_ara_*). Bars represent mean ± SD of three replicates. (**B**) The same set of plasmids was transformed into PA14 *fleQ*::Tn. Swimming was measured on 0.3% LB agar containing gentamicin and 0.2% arabinose. Bars represent mean ± SD of three replicates.

To test whether the conservative G240V amino acid change is responsible for the apparent loss of function of FleQ_PA103_, site-directed mutagenesis was used to revert Valine_240_ to Glycine in FleQ_PA103_. The presence of the V240G substitution was confirmed by Sanger sequencing, and the resulting protein was named FleQ(V240G)_PA103_. PA103 expressing the “corrected” FleQ(V240G)_PA103_ allele swam as well as PA103 expressing FleQ_PAO1_, while bacteria expressing FleQ_PA103_ did not swim (Figure 3A). These results confirm that the single conservative amino acid change of Glycine240 to Valine abrogates FleQ function. The same series of plasmids was also introduced into a PA14 *fleQ*::Tn mutant. Again, the FleQ_PA103_ allele did not complement the mutant for swimming, but either FleQ_PAO1_ or FleQ(V240G)_PA103_ could do so (Figure 3B).

**Figure 4:**
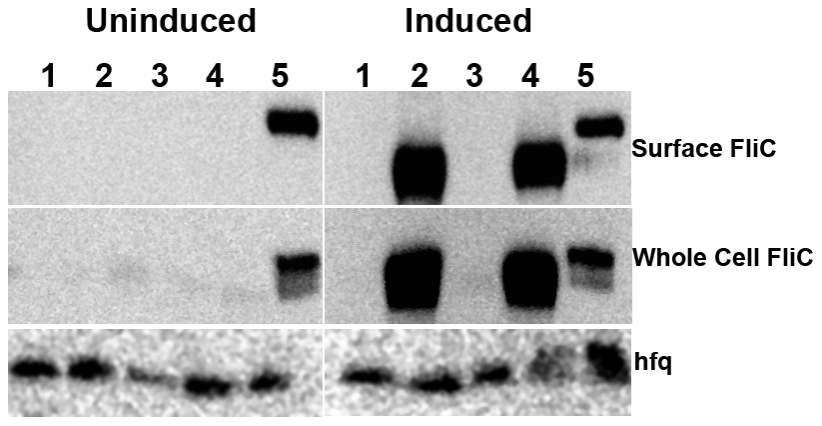
PA103 assembles flagella upon expression of corrected FleQ(V240G)_PA103_. Western blots of surface-associated **(upper panel)** and total flagellin **(middle panel)** were prepared from bacteria grown in LB without (uninduced) or with (induced) 0.2% arabinose. Samples were normalized to total protein (as measured in the cell pellet) prior to SDS-PAGE and Western blotting. Whole cell pellets were also probed with anti-Hfq antiserum to confirm equal loading **(lower panel)**. Lanes 1-4 are PA103 carrying (1) empty pMQ72 plasmid, (2) FleQ_PAO1_, (3) FleQ_PA103_ and (4) FleQ(V240G)_PA103_. Lane 5: PAO1 carrying an empty pMQ72 plasmid. Note difference in electrophoretic mobility between a-type flagellin of PA103 and b-type flagellin of PAO1.

Restoration of swimming motility was accompanied by expression of surface associated and whole cell flagellin in PA103 expressing FleQ_PAO1_ and FleQ(V240G)_PA103_; in contrast, no surface flagellin was detected in PA103 carrying an episomal copy of FleQ_PA103_ or the vector alone (Figure 4). We also confirmed that the naturally-occurring FleQ mutation in PA103 was associated with decreased expression of FleQ-dependent transcripts. Expression of *fliL* and *flhA* in PAO1 and PA103 was measured by qRT-PCR. In line with our observations for flagellin expression, mRNA levels of *fliL* and *flhA* in PA103 were 9.5±4% and 1.6±0.7%, respectively, of those measured in PAO1. Expression of the “corrected” FleQ(V240G)_PA103_ in PA103 increased mRNA levels of *flhA* 22±5.6 fold as compared to PA103 carrying the empty vector.

Although FleQ_PA103_ did not support flagellar gene expression or swimming motility, over-expression of this non-functional allele in PAO1 did not alter swimming zone size of PAO1, suggesting that it has no dominant negative effect.

### Restoring flagellar expression in PA103 has no measurable effect on T3SS

**Figure 5:**
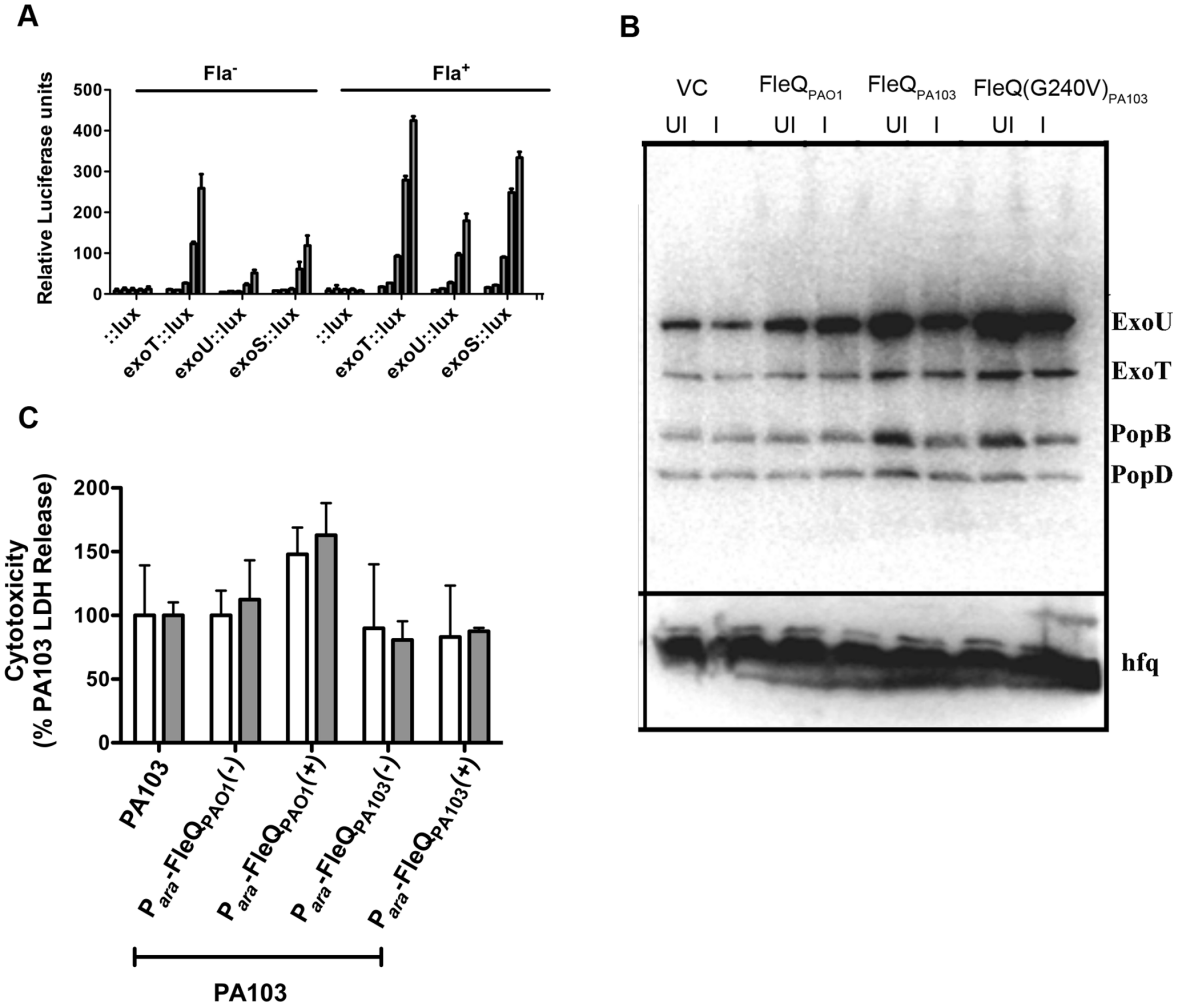
T3SS expression by PA103 is not altered when flagellar assembly is restored. (A) PA103 carrying *lux* transcriptional reporters for the *exoU*, *exoS* or *exoT* promoters, or a promoterless control, was transformed with P*_ara_*-FleQ(V240G)_PA103_ (Fla^+^) or empty vector (Fla^-^). RLU (luminescence/OD_600_) was measured hourly (0–4 h) after diluting bacteria into T3SS inducing media (MinS+NTA). Bars represent the mean ± SD of triplicate samples, and the experiment was independently reproduced three times. (**B**) Western blot of secreted T3SS effectors. Culture supernatants of PA103 carrying pMQ72 (VC), FleQ_PAO1_, FleQ_PA103_ or FleQ(V240G)_PA103_ were prepared from bacteria grown in MinS+NTA with (induced) or without (uninduced) 0.2% arabinose. Each lane was loaded with supernatant equivalent to 15 µg total protein, as measured in the cell pellet. ExoU, ExoT, PopB and PopD were detected by Western blotting with polyclonal antiserum. Cell pellets were immunoblotted with anti-Hfq antiserum to confirm equal loading. (**C**) Cytotoxicity toward HeLa cells as measured by LDH release. HeLa cells were infected with bacteria (MOI = 10) in triplicate and sampled at 1 h (white bars) and 2 h (grey bars) post-infection. Values are normalized to cytotoxicity caused by PA103 at each time point. The assay was repeated three times, and bars show the mean ± S.D. of an assay, which is representative of three independent experiments.

Soscia *et al*. previously observed an inverse relationship between flagellar assembly and Type 3 secretion system (T3SS) expression in *P. aeruginosa* [29]. We therefore tested whether restoration of flagellar assembly in PA103 altered the expression of genes encoding the T3SS effectors ExoU, ExoT or ExoS, using *lux* transcriptional reporters constructed for each of these promoters [30]. Contrary to expectations, we did not observe decreased transcriptional reporter activity when flagellar assembly was induced by the expression of FleQ(V240G)_PA103_ (Figure 5A). We also examined secretion of T3SS effectors by Western blot analysis, and did not observe any consistent changes in T3SS effector secretion between PA103 strains expressing functional (FleQ_PAO1_, FleQ(V240G)_PA103_) or non-functional (FleQ_PA103_, vector control) alleles of FleQ (Figure 5B). Lastly, T3SS-dependent cytotoxicity of bacteria expressing either FleQ_PAO1_ or FleQ_PA103_ toward HeLa cells was assayed by LDH release; bacteria were centrifuged onto cells after inoculation to minimize any effect of motility on efficiency of infection. Again, flagellated PA103 showed greater cytotoxicity in this assay than either aflagellate construct (Figure 5C). Thus, restoration of flagellar assembly to PA103 did not appreciably diminish T3SS expression or T3SS-dependent phenotypes *in vitro*.

### Increased clearance of Fla+ PA103 *in vivo*

Bacterial flagellin is a potent activator of the innate immune system, as both surface receptors (TLR5) and intracellular receptors (mNAIP5) respond to its presence. We tested whether flagellated PA103 would elicit an altered immune response during the course of infection or show differences in bacterial clearance, using a murine acute pneumonia model that is well-characterized for PA103 [31], [32]. For these experiments, the corrected *fleQ(V240G)_PA103_* gene was recombined onto the PA103 chromosome in place of the endogenous *fleQ* gene by allelic exchange, generating Fla^+^ PA103. Restoration of surface associated flagella (Figure S2A) and swimming motility were confirmed for this strain (Figure S2B). The specific growth rate (μ_max_) for Fla^+^ PA103 in LB was determined and found to be 1.5±0.2 h^−1^; this did not differ from that measured for wild-type PA103 in LB (1.4±0.2 h^−1^).

**Figure 6:**
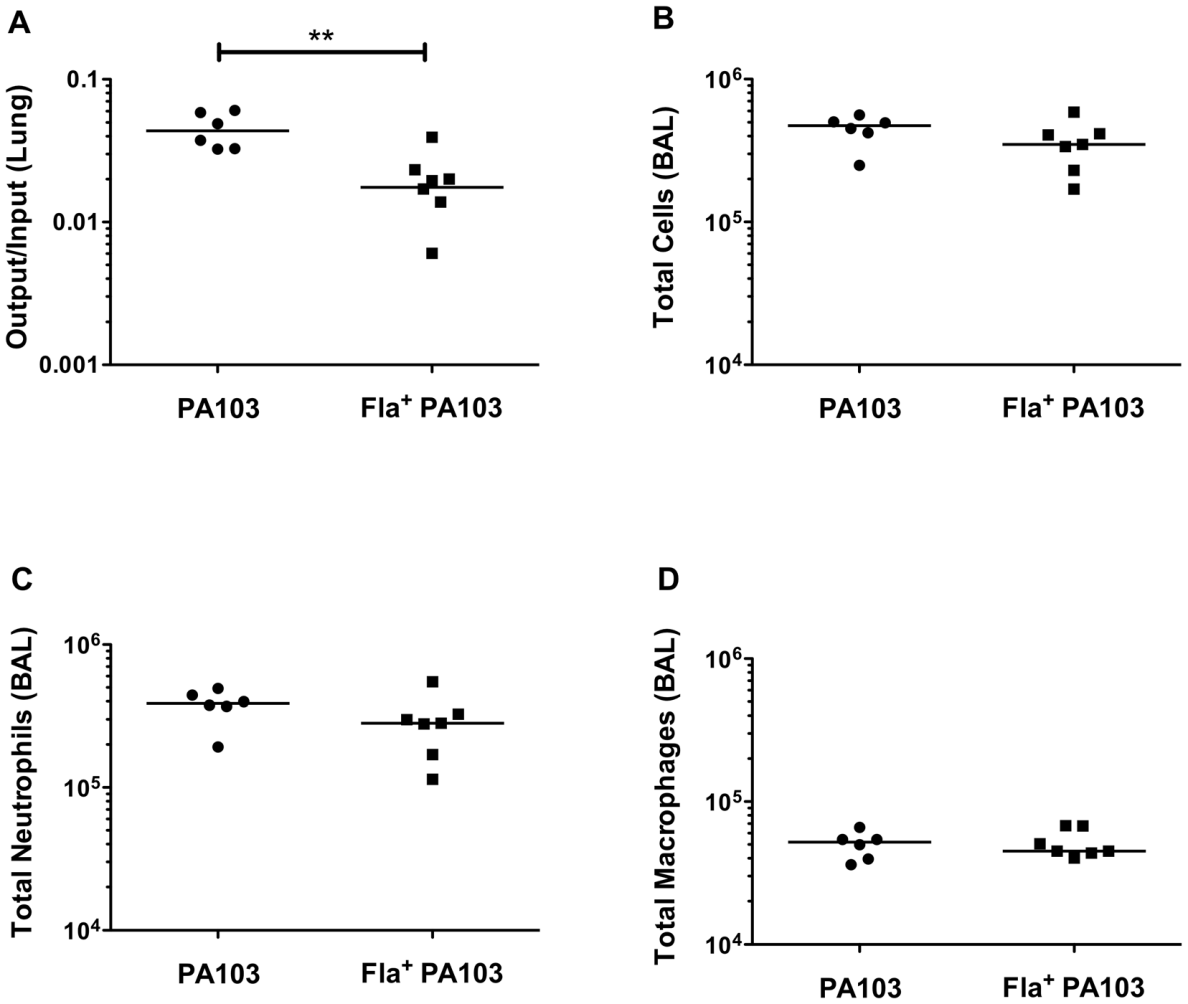
Flagellated bacteria are cleared more efficiently in mouse lung. C57BL/6 mice were intranasally infected with *P. aeruginosa* strain PA103 or *fla* ^+^ PA103, then sacrificed 6 hpi. (**A**) Lung bacterial burden expressed as number of bacteria recovered per lung (output) divided by measured inoculum administered (input). Each symbol represents an individual animal; the line indicates the geometric mean for each group. (**B**) Total cells, (**C**) neutrophils and (**D**) macrophages were counted in BAL. Each symbol represents an individual animal; lines indicate medians. Log-transformed data were analyzed using Mann-Whitney test (**, p<0.01).

**Figure 7:**
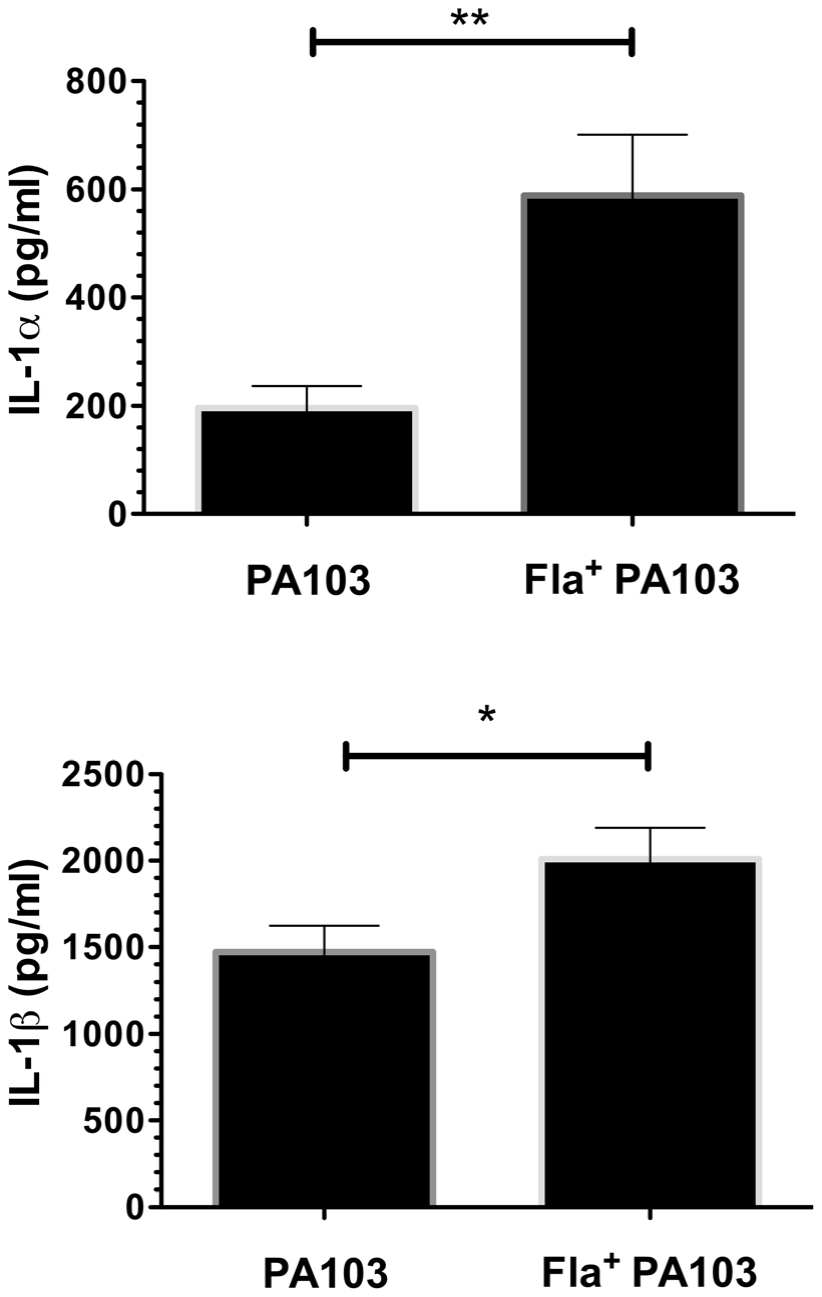
Fla*^+^* PA103 elicits more IL-1 cytokines than aflagellate (wild-type) PA103. BAL fluid was collected 6 hpi, and IL-1α (**A**) and IL-1β (**B**) were measured by sandwich ELISA. Bars indicate mean ± S.E.M. for groups of mice (n = 6-7); these were compared by t-test (*, *p*<0.05; **, *p*<0.01).

Mice were intranasally infected with 5×10^5^ cfu of PA103 or Fla^+^ PA103. At 6h post-infection both groups of mice showed the typical recruitment of neutrophils to the airways, a response that can be seen as early as 2h post-infection and which peaks ca. 12h post-infection [31], [32]. Although the number of neutrophils and macrophages recruited to the airway did not differ between the two groups of animals, Fla^+^ PA103 was recovered in lower numbers from mouse lungs at this time point (Figure 6). We also measured IL-1α (Figure 7A) and IL-1β(Figure 7B) concentrations in BAL fluid, and found higher levels of both cytokines in mice infected with the Fla^+^ PA103 strain.

## Discussion

The basis for PA103's inability to swim was first examined some 30 years ago[26], and has remained unresolved. We describe a naturally occurring mutation in PA103 *fleQ*, the gene encoding the master regulator of flagellar biosynthesis, that abrogates function of this transcriptional regulator and accounts for the aflagellate phenotype of PA103, a virulent acute pneumonia isolate. Reversion of the single conservative amino acid change (G240V) resulting from this mutation is sufficient to restore flagellar assembly and swimming motility to PA103. The amino acid substitution in FleQ_PA103_ does not appear to destabilize the protein, which is detected in comparable steady-state amounts in lysates of PA103 and PAO1. Rather, the mutation interferes with FleQ's function as a transcriptional activator. Amino acid 240 lies immediately upstream of the conserved hhhhDE motif within the Walker B box of FleQ. Mutational analysis of FleQ and homologous EBPs has demonstrated that this region of the protein is involved in hydrolyzing ATP, while Walker A box amino acids are responsible for nucleotide binding [15], [18]. Disruption of FleQ ATPase activity, which is thought to power remodelling of the RNAP complex and template loading, significantly reduces its ability to activate transcription, as shown by the effects of two physiological inhibitors, c-di-GMP and FleN, on flagellar gene expression.

Swimming motility has been correlated with *P. aeruginosa* virulence in murine models and in human infection [33]–[35]. Flagellar motility promotes bacterial dissemination and is necessary for initial surface colonization and biofilm formation; at the same time, flagellin is a potent immunogen and is recognized by several receptors of the innate immune system, e.g. TLR5 and NAIP5/NLRC4. *P. aeruginosa* bacteria exhibiting flagellar motility are taken up more efficiently by phagocytes than non-motile organisms, and elicit more robust innate immune responses during infection [36]. Thus, it is not surprising that bacteria can modulate the expression of flagellin in the host by a number of mechanisms that include both programmed switches into non-motile lifestyles [37] as well as regulated responses to immune system products, such as neutrophil elastase [38]. Although it is not possible to determine why a loss-of-function FleQ mutation was selected in a clinical isolate such as PA103, we did test whether restoring flagellar expression had any measurable effects on *in vitro* or *in vivo* virulence of this strain.

An earlier report by Soscia et al. suggested that T3SS activity and flagellar assembly are inversely related, as a PAO1*fliC* mutant showed increased Type 3 effector secretion relative to its isogenic wild-type parent [29]. Since PA103 secretes more T3SS effectors than 231 of 237 unique clinical isolates tested by quantitative ELISA [34], we investigated whether this was related to its lack of flagellar assembly. However, the restoration of flagellar assembly that accompanied FleQ(V240G)_PA103_ expression had no measurable effect on T3SS effector transcription, secretion, or activity in cell-based assays. It is possible that the phenotype observed by Soscia and colleagues is specific to the loss of *fliC* expression in a wild-type FleQ background, and requires expression of other FleQ-dependent genes. Alternatively, T3SS activity in PA103 may be determined by regulatory inputs other than the absence or presence of flagella.

Many studies have examined the consequences of a loss of flagellar motility or expression on virulence; we took advantage of our findings to test how restoration of flagellar expression would affect PA103 fitness in an acute pneumonia model. We observed that Fla^+^ bacteria were cleared more efficiently than Fla^-^ PA103. Our *in vitro* data suggest that this is not likely the result of decreased T3SS expression by the Fla^+^ strain. Instead, our observations support the findings of Berwin and colleagues, who noted that motile *P. aeruginosa* (PA14) elicit higher levels of inflammasome activation *in vitro* and elevated IL-1β responses *in vivo* following peritoneal infection as compared to non-motile mutants [36]. We likewise observed higher IL-1β and IL-1α levels in BAL fluid from lungs infected with Fla^+^ PA103 than the Fla^-^ strain. Both Fla^+^ and Fla^-^ infections elicited robust neutrophil recruitment to the airways, but Fla^+^ bacteria were cleared more efficiently. As non-flagellated *P. aeruginosa* mutants are more resistant to phagocytosis than motile counterparts [39], aflagellate PA103 may benefit not only by eliciting a diminished or delayed pro-inflammatory IL-1 host response, but also by evading clearance by resident and recruited phagocytes.

Sequencing of *P. aeruginosa* clinical isolates from chronically infected patients has demonstrated that many of the virulence factors essential for the establishment of infection are mutationally inactivated in these highly adapted strains. It is not clear if these genes are lost in the absence of positive selection, or if their expression confers decreased fitness upon bacteria colonizing the CF airways. Environmental *P. aeruginosa* strains and those associated with acute infections are much more likely to express virulence factors such as motility organelles and protein secretion systems, and thus it is of interest that a hypervirulent strain such as PA103 is aflagellate. Restoring flagellar assembly and swimming motility to this strain did not have the anticipated negative effect on its expression and secretion of T3SS effectors, but did increase both host inflammatory responses to infection and bacterial clearance, revealing the “cost” of possessing flagella in the setting of acute infection.

## Materials and Methods

### Ethics statement

All animal work was conducted according to relevant national and international guidelines. Protocols for all animal studies were approved by the Yale Institutional Animal Care and Use Committee.

### Bacterial strains and growth conditions

Strains and plasmids used in this study are listed in Table 2. *Escherichia coli* was cultured in Luria Broth (LB) and *Pseudomonas aeruginosa* was cultured in LB or Vogel–Bonner minimal (VBM) media [40] with antibiotics as required at the following concentrations: gentamicin, 15 µg ml^−1^ for *E. coli* or 100 µg ml^−1^ for *P. aeruginosa*; ampicillin, 100 µg ml^−1^ for *E. coli*; carbenicillin, 200 µg ml^−1^ for *P. aeruginosa*. To induce T3SS expression, *P. aeruginosa* was grown in MinS medium containing nitriloacetate (NTA) at 37°C with aeration [41]. Chemically competent *E. coli* DH5α was used for transformations (Invitrogen) and *P. aeruginosa* was transformed by electroporation [42]. All bacterial strains were stored at −80°C as 15% (v/v) glycerol stocks. Growth rates of *P. aeruginosa* cultures were determined as described [43].

**Table 2:** Strains and plasmids used in this study.

*Strains*	Description/Relevant genotype	Source
***E. coli***		
DH5α	*supE44 ΔlacU169*(Φ80d*lac*ZΔM15) *hsdR17 thi-1 relA1 recA1*	Invitrogen
S17.1	*thi pro hsdR recA* RP4-2 (Tc::Mu) (Km::Tn7)	[55]
***P. aeruginosa***		
PA103		[24]
PAO1		[56]
PA14*fleQ*::Tn		G. O'Toole
Fla^+^-PA103	PA103 with “corrected” FleQ(V240G)_PA103_ allele replacing endogeneous allele	This work
***Plasmids***		
pMQ72	Expression vector; Gm^r^ URA3 P_BAD_; *ara*C	[57]
pUCP-SK	Expression vector; Ap^r^ (Cb^r^)	[58]
pEX18-Gm	Allelic exchange vector; Gm^r^ *sacB oriT*	[59]
pEX-*fleQ*(V240G)_PA103_	“Corrected” FleQ(V240G)_PA103_ cloned in pEX18 vector; Gm^r^	This work
P*_lac_*-FleQ_PAO1_	FleQ_PAO1_ cloned in pUCP-SK under *lac* promoter; Cb^r^	This work
P*_ara_*- FleQ_PAO1_	FleQ_PAO1_ cloned under pBAD promoter in pMQ72; Gm^r^	This work
P*_ara_*- FleQ_PA103_	FleQ_PA103_ cloned under pBAD promoter in pMQ72; Gm^r^	This work
P*_ara_*- FleQ(V240G)_PA103_	FleQ(V240G)_PA103_ cloned under pBAD promoter pMQ72; Gm^r^	This work

### Plasmid and Strain Construction

The *fleQ* gene was amplified from PAO1 and PA103 genomic DNA using primers FleQF and FleQR (Table 3). Amplicons were digested with EcoRI and HindIII, then cloned into pMQ72 and pUCP-SK for expression in *P. aeruginosa*; all constructs were confirmed by nucleotide sequencing (Keck Sequencing Facility, Yale). Arabinose was used at a concentration of 0.2% to induce expression of genes from the pMQ72 vector.

**Table 3:** Primers used in this study.

Primer Name	Primer Sequence (5′-3′)
FQRTF1	GCGCGAAACCAAACTCTTGCTGAT
FQRTR1	TCGCCGAGGAAGTTGAGAATGACT
FliLRTF	GCTGTTCTCGAGTCAGAGTTT
FliFRTR	TTGGCCGGTTTCCTTCTT
FlhARTF	CGGACAACAAGCAAGTCACCATCG
FlhARTR	ATCGGCGGCGAAGAAGCGTTTGAC
FQV240GF	CGAGCTGGCCAACGGCGGCACCCTGTTCCTCGACG
FQV240GR	CGTCGAGGAACAGGGTGCCGCCGTTGGCCAGCTCG
FleQF	GACGAATTCGATGCAAGGCAGCTGATCAAAATGTGGCGCG
FleQR	GAAAATCAAAAGCTTGCGAAACGACCTGTCAATCATC

FleQ_PA103_ was mutated to FleQ(V240G)_PA103_ by changing Valine 240 to Glycine (nt: T718G) according to the QuikChange Site-Directed Mutagenesis kit protocol (Stratagene), using primers FQV240GF and FQV240GR (Table 3). FleQ_PA103_ cloned in pUCP was used as a template for the mutagenesis reaction, and the introduction of only the desired mutation was confirmed by nucleotide sequencing. FleQ(V240G)_PA103_ was then subcloned into pMQ72 and pEX18-Gm^R^ as a EcoR1-HindIII fragment. The V240G mutation was introduced into the endogenous chromosomal copy of *fleQ* in PA103 via allelic exchange [44]. Briefly, pEX-*fleQ*(V240G)_PA103_ was transformed into *E. coli* S17-1 and was mobilized into *P. aeruginosa* by mating. Selection of double recombinants was carried out on 10% sucrose and mutant constructs were confirmed by sequencing and phenotypic characterization.

### RNA isolation and quantitative real time PCR

Bacterial RNA was isolated from early log-phase cells (OD_600_ = 0.4) as previously described [45]. cDNA was synthesized from 1 µg RNA using iScript cDNA Synthesis kit (BioRad). For qRT-PCR, 1 µl of cDNA was added to SYBRGreen qPCR mastermix (BioRad) containing 500 nM of forward (-RTF) and reverse (-RTR) primers (Table 3). Transcript levels of all genes tested were normalized to transcript levels of the ribosomal gene, *rplU*. Data presented are the mean ± S.D. of triplicate determinations from two independent experiments.

### Whole Genome Sequencing and Sequence analysis

Bacterial genomic DNA was prepared for sequencing on the Illumina MiSeq by the Yale Center for Genomic Analysis using the TruSeq DNA LT Sample Prep Kit (Illumina, Inc. San Diego, CA USA). Steps were performed as described in the TruSeq DNA Sample Preparation Guide (#15026486 Rev.C, July 2012). Purified libraries were barcoded, pooled and sequenced on the MiSeq using a 2×250 paired-end protocol. Initial basecalls were converted to fastq files using MiSeq CASAVA software suite, de-multiplexed and clipped for adaptors. Sequences were examined using FastQC (Galaxy). Reads were mapped to the *P. aeruginosa* PAO1 reference genome with the BWA (v. 0.6.2) software package using default parameters [46], [47]. Bowtie (v. 2.1.0) was used to align reads [48], and SNPs were called using the Samtools mpileup [49]. We also carried out a de novo assembly with Velvet (v. 1.1.04) [50] using default parameters, and *k*-mer length of 35. Contigs containing flagellar genes were identified with nucleotide BLAST (blastn) against *Pseudomonas aeruginosa* genomes. Restriction enzyme site analyses of DNA sequences and multiple-sequence alignments (with CLUSTAL W) were performed using the BioEdit sequence alignment editor (version 7.0; Tom Hall) or MacVector (Version 11.1). Secondary structure analysis of the protein sequence was carried out using NCBI CD Search [51]. Nucleotide sequences for regions encoding the flagellar genes *flgB-fliJ* and *fliK-motD* have been deposited to Genbank (KJ411884 and KJ411885).

### Swimming Assay

Swimming assays were performed by spotting 5 µl of an overnight bacterial culture on 0.3% LB agar plates supplemented with antibiotics (and 0.2% arabinose as appropriate). The swimming zone was measured after overnight incubation at 30°C. Plates were photographed with an Image Station 2000R (Kodak).

### Surface expression of flagellin

Surface flagella were harvested as previously described [52]. Briefly, *P. aeruginosa* strains were grown at 37°C in LB (with antibiotics as required), harvested by centrifugation, and resuspended in 50 mM sodium phosphate (pH 7.0) containing 10 mM MgCl_2_. Flagella were sheared by blending bacterial suspensions for 20 seconds in a Waring blender. Flagellar detachment was monitored by viewing cells under the microscope for loss of swimming. Samples were centrifuged at 12,000×*g* for 30 min at 4°C to pellet cell bodies. The supernatants containing sheared flagella were separated by SDS-PAGE and blotted to PVDF. Flagellin was detected using anti-FliC antiserum (1∶4000) [53]. A goat antirabbit IgG horseradish peroxidase conjugate was used as a secondary antibody (1∶4000, Bio-Rad). Detection was performed using ECL substrate (100 mM Tris-HCL pH 8.5, 250 mM luminol, 90 mM coumarate, 0.009% H_2_O_2_), and blots were visualized and anlysed using an Image Station 2000R running Carestream MI Software Version 5.0.2 (Kodak). Protein in cell pellets was determined by BCA assay (Pierce) and supernatant equivalent to 25 µg was loaded on the gel for analysis. Rabbit polyclonal antiserum generated against *P. aeruginosa* Hfq was used to confirm equal loading of protein samples.

### T3SS secretion assay

Single bacterial colonies were used to inoculate 10 mL MinS supplemented with antibiotics and arabinose, as appropriate. Cultures were grown with shaking at 37°C for 12–14 h. Cells were pelleted, resuspended in buffer [20 mM Tris.Cl, pH 8.0, 137 mM NaCl, 10% glycerol, 1% Triton X-100, 2 mM EDTA and protease inhibitors] and lysed by sonication. Total protein in cell pellets was determined using BCA Assay (Pierce). Supernatants were normalized based on total protein of corresponding cell pellets, separated by SDS-PAGE and blotted to PVDF. Secreted T3SS proteins were detected using anti-T3SS polyclonal antiserum (1∶10,000) [54], followed by incubation with secondary antibody and ECL substrate as described above.

### Luciferase constructs and the luminescence reading

Bacterial strains were grown in LB with aeration at 37°C for 14–16 h. Cultures were centrifuged and bacterial pellets were resuspended and washed twice in MinS containing NTA. Samples were subcultured 1∶100 into 10 ml of MinS and incubated at 37°C with shaking. Luminescence was measured at 1 h intervals starting at time zero using a luminometer (Turner Designs, Sunnyvale, CA). Relative Luciferase Units (RLU) was calculated by dividing luminescence by the optical density (OD_600_) of each culture at time of sampling.

### LDH release assay

HeLa cells (ATCC) were cultured in DMEM (Gibco) supplemented with 10% heat-inactivated fetal calf serum (Atlanta Biologicals) and 10 mM L-glutamate in a 5% CO_2_ humidified incubator at 37°C. For LDH assays, 2×10^5^ cells/well were seeded 24 h prior to infection into 24-well tissue culture plates. Overnight bacterial cultures in LB (with antibiotics and arabinose if required) were subcultured into fresh media, grown to early exponential phase, and diluted in DMEM plus 1% FCS. HeLa cells were washed with PBS prior to infection, which was initiated by centrifuging bacteria (MOI = 10) onto Hela cell monolayers (1000×*g* for 5 minutes at RT). LDH released into culture media at different time points was measured according to manufacturer's instructions (Takara Bio).

### Mouse infections

C57Bl/6 mice were purchased from the National Cancer Institute (NIH) and housed in isolator cages under specific pathogen-free conditions. Groups of 8 wk-old female mice were intranasally infected with ca. 5×10^5^ CFU of early log-phase bacteria as described previously [31]. Mice were euthanized 6 h post-infection. Bronchoalveolar lavage fluid was collected and processed for cytokine assays and differential counts as previously described [31]. Lungs were aseptically removed, homogenized in PBS +0.1% Triton-X100, and passed through a sterile screen to obtain single cell suspensions. Samples were serially diluted and plated on VBM agar. Colonies were counted after 36 h incubation at 37°C.

### Measurement of cytokines

IL-1α and IL-1β in BAL fluid were measured by sandwich ELISA (R&D Systems) according to the manufacturer's protocol.

### Statistical analysis

Data were analyzed in GraphPad Prism (v5.0). Mann-Whitney test was used for pair-wise comparisons. *p* values<0.05 were considered statistically significant.

## Supporting Information

Figure S1 **Sequence analysis of FleQ.** (**A**) **Domain structure of FleQ.** Conserved domains of FleQ were identified using the NCBI CD search tool and include an amino-terminal FleQ domain, an ATP binding domain (AAA) containing Walker A and Walker B boxes, and a helix-turn-helix (HTH) domain. (**B**) **Multiple sequence alignment of FleQ from Pseudomonads.** Analysis includes *P. aeruginosa* strains LESB58, UCBPP-PA14, PAO1, PAK, PA103, and *P. fluorescens*, *P. putida, P. syringae* and *P. stutzeri*. (**C**) **Multiple Sequence alignment of FleQ_PAO1_ and FleQ_PA103_ with homologous transcriptional regulators from other organisms.** Accession numbers corresponding to the proteins used in this analysis are as follows: FleQ [*Legionella pneumophila*], CAD97470; FlbD [*Caulobacter crescentus*], AAA23039; NifA [*Klebsiella pneumoniae*], YP_002237549; FleQ [*Vibrio cholerae*], WP_001961246; FleQ [*Xanthomonas campestris*], YP_363745 and FleR [*Pseudomonas aeruginosa*], NP_249790. The residue corresponding to FleQ_PAO1_ G240 is boxed.
(TIF)

Figure S2 **Fla^+^ PA103 expresses functional surface flagella.** (A) Western blot of surface flagellin preps and whole cells prepared from overnight cultures of PA103, PAO1 or Fla^+^ PA103; flagellin was detected with anti-FliC polyclonal rabbit serum. Whole cell lysates were also probed with anti-Hfq antiserum to confirm equal loading. (**B**) Swimming zones of *P. aeruginosa* strains as measured on 0.3% LB agar after incubation for 16 h at 30°C. Bars represent mean ± S.D. of three independent experiments.
(TIFF)
