# Supplementary figures and images for: A Conservative Amino Acid Mutation in the Master Regulator FleQ Renders Pseudomonas aeruginosa Aflagellate
Source: PLoS One. 2014 May 14;9(5):e97439. doi: 10.1371/journal.pone.0097439 (PMC4020848; doi:10.1371/journal.pone.0097439)

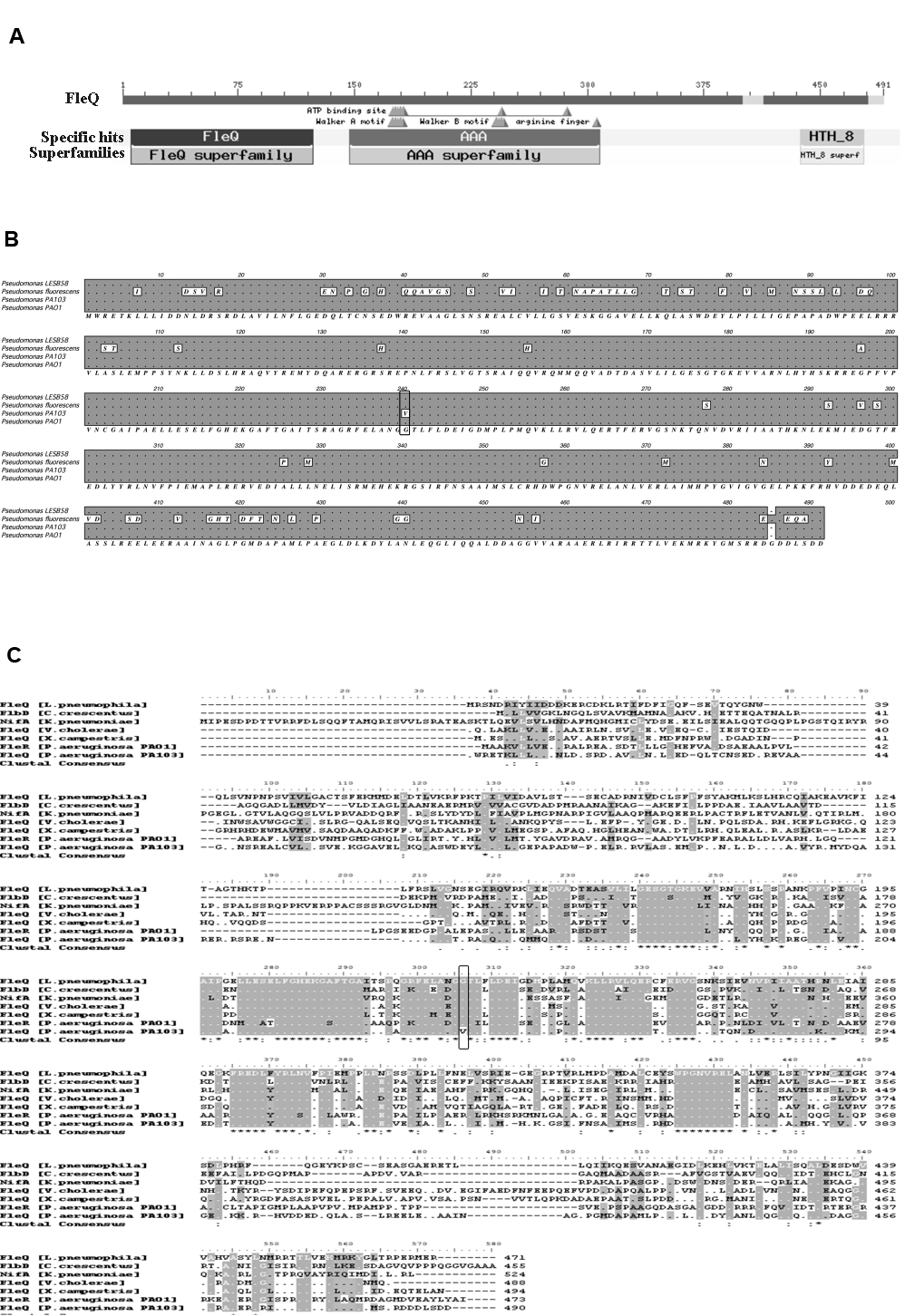

Supplement: Figure S1 — Sequence analysis of FleQ. (A) Domain structure of FleQ. Conserved domains of FleQ were identified using the NCBI CD search tool and include an amino-terminal FleQ domain, an ATP binding domain (AAA) containing Walker A and Walker B boxes, and a helix-turn-helix (HTH) domain. (B) Multiple sequence alignment of FleQ from Pseudomonads. Analysis includes P. aeruginosa strains LESB58, UCBPP-PA14, PAO1, PAK, PA103, and P. fluorescens, P. putida, P. syringae and P. stutzeri. (C) Multiple Sequence alignment of FleQPAO1 and FleQPA103 with homologous transcriptional regulators from other organisms. Accession numbers corresponding to the proteins used in this analysis are as follows: FleQ [Legionella pneumophila], CAD97470; FlbD [Caulobacter crescentus], AAA23039; NifA [Klebsiella pneumoniae], YP_002237549; FleQ [Vibrio cholerae], WP_001961246; FleQ [Xanthomonas campestris], YP_363745 and FleR [Pseudomonas aeruginosa], NP_249790. The residue corresponding to FleQPAO1 G240 is boxed. (TIF) [file pone.0097439.s001.tiff]

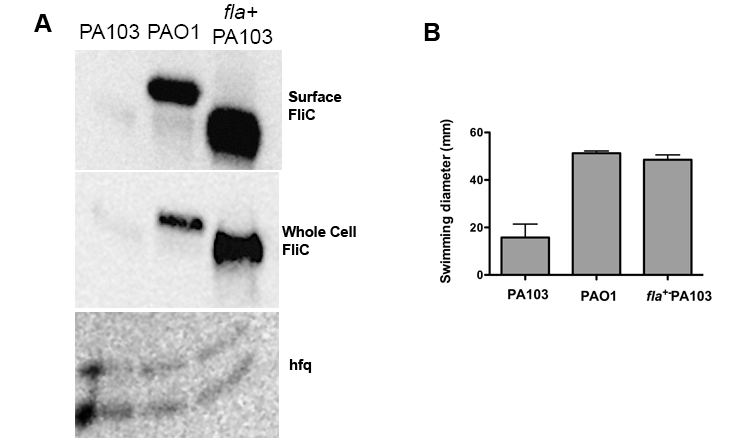

Supplement: Figure S2 — Fla+ PA103 expresses functional surface flagella. (A) Western blot of surface flagellin preps and whole cells prepared from overnight cultures of PA103, PAO1 or Fla+ PA103; flagellin was detected with anti-FliC polyclonal rabbit serum. Whole cell lysates were also probed with anti-Hfq antiserum to confirm equal loading. (B) Swimming zones of P. aeruginosa strains as measured on 0.3% LB agar after incubation for 16 h at 30°C. Bars represent mean ± S.D. of three independent experiments. (TIFF) [file pone.0097439.s002.tiff]
